# Neuropeptide W facilitates chronic gastric ulcer healing by the regulation of cyclooxygenase and NF-κB signaling pathways

**DOI:** 10.1007/s10787-023-01403-w

**Published:** 2024-01-16

**Authors:** Sevil Arabacı Tamer, Kadriye Sezen Mermer, Ömer Erdoğan, Özge Çevik, Feriha Ercan, Cahit Bağcı, Berrak Ç. Yeğen

**Affiliations:** 1 Department of Physiology, Sakarya University School of Medicine, Sakarya, Turkey; 2 Department of Physiology, Marmara University School of Medicine, Istanbul, Turkey; 3 Faculty of Medicine, Department of Biochemistry, Aydın Adnan Menderes University, Aydın, Turkey; 4 Department of Histology & Embryology, Marmara University School of Medicine, Istanbul, Turkey

**Keywords:** Neuropeptide W, Acetic acid-induced gastric ulcer, Oxidative injury, Inflammation, Cyclooxygenases, Prostaglandins

## Abstract

**Aims:**

Putative beneficial effects of neuropeptide W (NPW) in the early phase of gastric ulcer healing process and the involvement of cyclooxygenase (COX) enzymes were investigated in an acetic acid-induced gastric ulcer model.

**Main methods:**

In anesthetized male Sprague–Dawley rats, acetic acid was applied surgically on the serosa and then a COX-inhibitor (COX-2-selective NS-398, COX-1-selective ketorolac, or non-selective indomethacin; 2 mg/kg/day, 3 mg/kg/day or 5 mg/kg/day; respectively) or saline was injected intraperitoneally. One h after ulcer induction, omeprazole (20 mg/kg/day), NPW (0.1 μg/kg/day) or saline was intraperitoneally administered. Injections of NPW, COX-inhibitors, omeprazole or saline were continued for the following 2 days until rats were decapitated at the end of the third day.

**Key findings:**

NPW treatment depressed gastric prostaglandin (PG) I2 level, but not PGE2 level. Similar to omeprazole, NPW treatment significantly reduced gastric and serum tumor necrosis factor-alpha and interleukin-1 beta levels and depressed the upregulation of nuclear factor kappa B (NF-κB) and COX-2 expressions due to ulcer. In parallel with the histopathological findings, treatment with NPW suppressed ulcer-induced increases in myeloperoxidase activity and malondialdehyde level and replenished glutathione level. However, the inhibitory effect of NPW on myeloperoxidase activity and NPW-induced increase in glutathione were not observed in the presence of COX-1 inhibitor ketorolac or the non-selective COX-inhibitor indomethacin.

**Significance:**

In conclusion, NPW facilitated the healing of gastric injury in rats via the inhibition of pro-inflammatory cytokine production, oxidative stress and neutrophil infiltration as well as the downregulation of COX-2 protein and NF-κB gene expressions.

## Introduction

Peptic ulcer disease (PUD), a global problem with a lifetime risk of development, is characterized by denuded mucosa extending into the submucosa or muscularis propria of the stomach and proximal duodenum (Narayanan et al. 2018; Salari et al. 2022). PUD results from an imbalance between the protective (e.g., mucus, bicarbonate, prostaglandins, antioxidant system and cell proliferation) and destructive (e.g., gastric acid, pepsin, *Helicobacter pylori*, stress, nonsteroidal anti-inflammatory drugs (NSAIDs) and alcohol) factors (Laine et al. 2008; Sverdén et al. 2019), while impaired gastric blood flow, oxidant damage, apoptosis, production of reactive oxygen metabolites and pro-inflammatory cytokines (e.g., tumor necrosis factor (TNF)-α, interleukin (IL)-6 and IL-1β) along with alterations in the expressions of nuclear factor kappa B (NF-κB) and prostaglandins (PGI2 and PGE2) aggravate gastric injury (Arabaci Tamer et al. 2021; Kolgazi et al. 2017; Tamer et al. 2022). Experimental gastric ulcer induced by acetic acid, which affects the entire gastric mucosa and submucosa and penetrates the muscularis mucosa, mimics the morphological and clinical findings observed in peptic ulcer patients (Arabacı Tamer et al. 2020; Okabe and Magase 2005), and therefore it is widely used as a chronic gastric ulcer model to investigate putative anti-ulcer alternative medications (Oyetayo et al. 2022; Wang et al. 2022).

The novel peptide neuropeptide W (NPW), which has 23 (NPW23) or 30 (NPW30) amino acid residues, was proposed as a mediator in controlling the neuroendocrine response to stress, because stress induction stimulates NPW-containing hypothalamic neurons and central administration of NPW in rats activates the hypothalamus-pituitary adrenal (HPA) axis (Niimi and Murao 2005). Regarding its expression in the hypothalamus and brain stem, NPW suppresses neuropathic pain and regulates food intake and energy homeostasis (Fujii et al. 2002; Mondal et al. 2003; Takenoya et al. 2012) via its orphan G-protein-coupled GPR7 (NPBWR1) and GPR8 (NPBWR2) receptors (Shimomura et al. 2002; Takenoya et al. 2012). Exogenously administered NPW modulates blood pressure and cardiovascular functions by its central as well as direct vascular effects (Ji et al. 2015; Pate et al. 2013; Yu et al. 2007). Moreover, we have recently reported that peripherally administered NPW alleviates colonic inflammation, and protects against sepsis-induced oxidative multiorgan injury (Atici et al. 2022) and stress-induced acute gastric ulcer (Arabacı Tamer et al. 2023). However, underlying mechanisms for the therapeutic action of NPW on the healing of gastric injury were not evaluated yet.

The damaging effect of NSAIDs on the gastroduodenal mucosa mainly occurs by systemic suppression of constitutively produced cyclooxygenase-1 (COX-1)-derived prostaglandins (PG) (Bereda 2022). Depleted mucosal PG content consequently results in decreased mucus and bicarbonate generation, reduces mucosal blood flow and inhibits cell proliferation, disrupting the integrity of gastric mucosa. Thus, COX-1 offers a physiological protection and plays an important role in the functional responses of the stomach to an injurious insult (Yandrapu and Sarosiek 2015). On the other hand, COX-2 is an early response gene, which is upregulated by cytokines, endotoxins, and mitogens upon an inflammatory process (Morita 2002). During the healing process of chronic gastric ulcers, both COX-1 and COX-2 isoforms contribute to mucosal defense by maintaining mucosal integrity and decreasing acid secretion (Tache and Perdue 2004; Takeuchi and Amagase 2018). PUD is a multifactorial disease and there is an ongoing demand for the identification of new therapeutic agents for its management (Zatorski 2017). Based on the aforementioned studies, our aim was to elucidate whether NPW could have beneficial effects against acetic acid-induced gastric ulcer, and to examine involvement of COX enzymes for its action in the early phase of the gastric ulcer healing process.

## Materials and methods

### Animals

Male Sprague–Dawley rats (12 weeks old; *n* = 56), supplied by Sakarya University Animal Center (SÜDETAM), were kept in an air-conditioned room with 12-h light and dark cycles, where the temperature (22 ± 2 °C) and relative humidity (65–70%) were kept constant. The animals were fed a standard pellet with free access to food and water, except for the withdrawal of food for an overnight fasting before gastric ulcer induction. The experiments were performed in compliance with Turkish law on the use of animals in experiments, and the principles and guidelines developed by the New York Academy of Sciences were followed. The experimental procedures were approved by the Sakarya University Animal Care and Use Committee (approval number: 38; date: 01/12/2021).

### Experimental procedure and ulcer induction

**Fig. 1:**
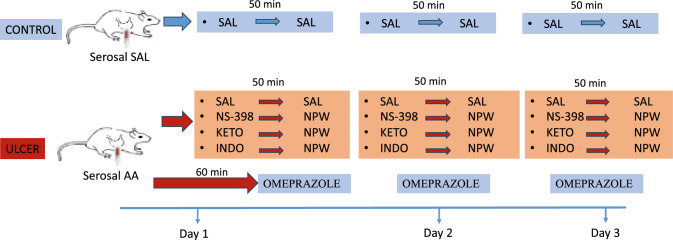
Timing diagram of the experimental protocol. Saline (SAL), neuropeptide W (NPW; 0.1 µg/kg/day), COX-2 inhibitor NS-398 (2 mg/kg/day), COX-1 inhibitor ketolorac (KETO; 3 mg/kg/day), non-selective COX-inhibitor indomethacin (INDO; 5 mg/kg/day) or omeprazole (20 mg/kg/day) were injected intraperitoneally following ulcer induction and were continued for the following 2 days

Gastric ulcer was induced using the method that was originally defined by Okabe and Pfeiffer (Okabe et al. 1971), who have reported the occurrence of chronic mucosal ulcers within 2–3 days after induction. After an overnight fasting, an upper abdominal midline incision was made under anesthesia (100 mg/kg ketamine and 10 mg/kg xylazine, intraperitoneally, i.p.). Using a 3-ml syringe, acetic acid solution (80%, vol/vol) was applied in a 0.5 ml volume onto the serosal surface of the gastric corpus, exposing the acid content on an area of 60-mm^2^. Following a 1-min contact, acetic acid was aspirated and gently washed out from the serosal surface with saline and the incision was closed. In the control groups, rats had the same surgical procedures, but a saline-containing syringe was put on the gastric serosa. Immediately after the gastric ulcer induction, COX-2-selective inhibitor NS-398 (2 mg/kg/day; Sigma-Aldrich, St. Louis, Missouri, USA), COX-1-preferring inhibitor ketorolac (3 mg/kg/day; Sigma-Aldrich, St. Louis, Missouri, USA), non-selective COX-inhibitor indomethacin (5 mg/kg/day; Sigma-Aldrich, St. Louis, Missouri, USA) or saline was injected i.p. (Fig. 1). Fifty minutes later, experimental groups were treated i.p. with either saline or NPW (0.1 μg/kg/day; Phoenix Pharmaceuticals). In our previous study, a dose–response assessment (0.1, 0.3, 1 and 5 μg/ kg) was performed to determine the effective dose of NPW on gastric injury [6], and consequently the 0.1 µg/kg dose of NPW was selected regarding its beneficial actions on ulcer scoring, gastric blood flow and oxidative parameters. As a positive control group, proton pump inhibitor omeprazole (20 mg/kg/day) was injected i.p. at 1 h of ulcer induction (Zewdu and Aragaw 2020). Injections of NPW, COX-inhibitors, omeprazole and saline were continued for the following 2 days. The doses of NPW (Tamer et al. 2022) and COX-inhibitors (Kolgazi et al. 2017) were selected based on our previous studies. At the end of the third day and one hour after the last injection, cardiac puncture was made under anesthesia to obtain blood and gastric tissue samples for biochemical, molecular and histopathological analyses.

### Measurement of gastric myeloperoxidase activity

After the homogenization step with 50 mM potassium phosphate buffer, myeloperoxidase (MPO) activity in the gastric tissues was measured based on H2O2-dependent oxidation of o-dianisidine.2HCl using a spectrophotometer (PG Instruments Ltd., UK) at 460 nm wavelength. MPO activity, which is an indicator of tissue neutrophil infiltration, was expressed as units per gram tissue (Tuğtepe et al. 2007).

### Determination of gastric malondialdehyde and glutathione levels

Gastric tissue samples were homogenized in trichloroacetic acid (10%, TCA) by an Ultra Turrax tissue homogenizer. In order to evaluate the degree of lipid peroxidation, malondialdehyde (MDA) levels were measured spectrophotometrically at 535 nm wavelength by observing TCA reagent formation and were presented as nmol MDA / gram tissue. Using the modified Ellman procedure, antioxidant glutathione (GSH) levels were determined spectrophotometrically at an absorbance value of 412 nm and the quantity of GSH was given as μmol/g tissue (Tuğtepe et al. 2007).

### Measurement of TNF-α, IL-1β and IL-10 levels in the serum and gastric tissues

Gastric tissue was homogenized in pH 7.4 PBS buffer containing 0.5% NP-40 (v/v) and protease inhibitor cocktail. Serum and gastric levels of the pro-inflammatory cytokines TNF-α (BT-Lab E0764Ra), IL-β (BT-Lab E0119Ra) and IL-10 (BT-Lab E0108Ra) were measured by using rat ELISA kits according to the manufacturer's procedure.

### Measurement of gastric prostaglandin E2 and prostaglandin I2 levels

Gastric tissue was homogenized in pH 7.4 PBS buffer containing 0.5% NP-40 (v/v) and protease inhibitor cocktail. Prostaglandin E2 (PGE2; Elabscience E-EL-0034) and PGI2 (Elabscience E-EL-0022) in the gastric samples were performed using ELISA kits according to the manufacturer's instructions. Protein concentrations in the gastric tissue samples were determined according to the Bradford method and prostaglandins were expressed as ng/mg protein or pg/mg protein, respectively.

### Gene expression analysis by reverse transcriptase-quantitative real-time PCR (RT-qPCR)

After the homogenization step by using a TissueLyser II (Qiagen, Hilden, Germany) in the gastric tissue, total RNA was isolated using a commercial kit (12183018A PureLink™ RNA Mini Kit, Thermo Fisher) in accordance with the manufacturer’s recommendations. Total RNA was measured using a NanoDrop 1000 Spectrophotometer (Fisher Thermo, Wilmington, DE, USA) and stored at -80° C. For the conversion of RNA to cDNA, cDNA synthesis with reverse transcriptase enzyme was performed using High-Capacity cDNA Reverse Transcription kit (4,368,814, Applied Biosystems, Foster City, California, USA).

Expression levels of NF-κB, COX-1, COX-2, and GAPDH were quantified by Quantitative Real Time-PCR (qPCR) assays in triplicate. Sybr green master mix (Applied Biosystems, A25742) for NF-κB, COX-1, COX-2 and GAPDH were used for performing qPCR reactions in ABI StepOne Plus detection system according to manufacturer’s instructions. Gene expression levels were calculated using 2 − ΔΔCt method with StepOne software version 2.3 for each sample. GAPDH was used as the endogenous control.

### Histopathological examination

For light microscopic investigations, gastric samples were fixed in a 10% formaldehyde solution, processed by routine paraffin embedding technique. Approximately 5-μm thick paraffin section of gastric tissues were mounted on slides and stained by hematoxylin and eosin for general histopathological evaluation. At least five similar areas in each section were examined under a photomicroscope (Olympus BX51, Tokyo, Japan). Histological assessment was made by a histologist (FE), who was blinded to group names. Histopathological evaluation was made semi-quantitatively with a maximum score of 12 for gastric tissues (0, none; 1, mild; 2, moderate; 3, severe). Histopathological criteria for the evaluation of gastric tissues were desquamation of surface epithelium, hemorrhage, focal necrosis and mucosal congestion, degeneration of glandular cells, and inflammatory cell infiltration (Arabacı Tamer et al. 2020).

### Statistical analysis

Using GraphPad Prism 9.3.0 (GraphPad Software, San Diego, CA, USA), one-way ANOVA followed by the Bonferroni multiple comparisons test was used to determine the level of statistical significance between experimental groups. All data were expressed as mean ± standard error. *p* < 0.05 was considered as statistically significant.

## Results

**Fig. 2:**
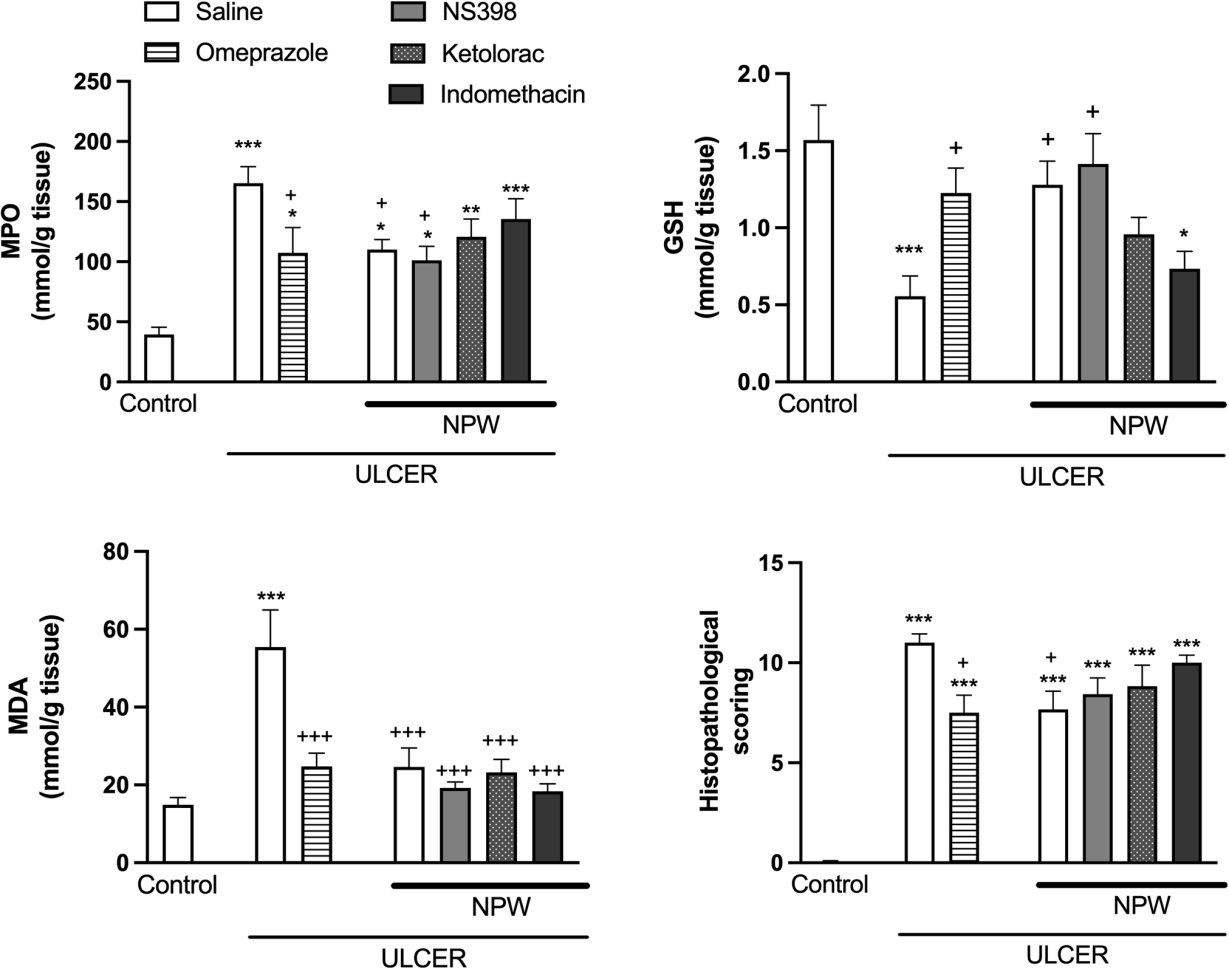
Myeloperoxidase (MPO) activity, malondialdehyde (MDA) levels, glutathione (GSH) content, and microscopic score in the gastric tissues of experimental groups. Acetic acid-induced ulcer groups were treated with either saline, omeprazole, neuropeptide W (NPW) + saline, NPW + cyclooxygenase (COX)-2 inhibitor NS-398, NPW + COX-1 inhibitor ketolorac and non-specific COX-inhibitor indomethacin for 3 days starting with the induction of acetic acid ulcer and were compared with the gastric tissues of control rats. Each group consisted of 8 rats. *p < 0.05, *p* < 0.01, ***p < 0.001, compared to control group; + *p* < 0.05, *p* < 0.001, compared to saline-treated ulcer group

Gastric levels of MDA, showing lipid peroxidation, as well as antioxidant GSH content were evaluated to determine the extent of oxidative injury, while gastric MPO activity was determined as an indicator of neutrophil infiltration to gastric tissue. Serosal application of acetic acid resulted in elevated MDA levels, MPO activity as compared to saline-treated control rats, while GSH content of gastric tissue was depleted (*p* < 0.001, Fig. 2). In omeprazole-treated ulcer group, as compared to saline-treated rats with ulcer, the gastric levels of MDA, and MPO activity were reduced and gastric GSH content was significantly elevated (*p* < 0.05). Similar to that observed in omeprazole-treated rats, treatment with NPW suppressed ulcer-induced increases in MPO activity and MDA level (*p* < 0.05) and replenished the antioxidant GSH level (*p* < 0.05). However, the inhibitory effect of NPW on MPO activity and NPW-induced increase in GSH content were not observed when NPW injections were preceded with either the selective COX-1 inhibitor ketorolac or the non-selective COX-inhibitor indomethacin, but the effects of NPW were not altered by the COX-2 inhibitor NS-398.

**Fig. 3:**
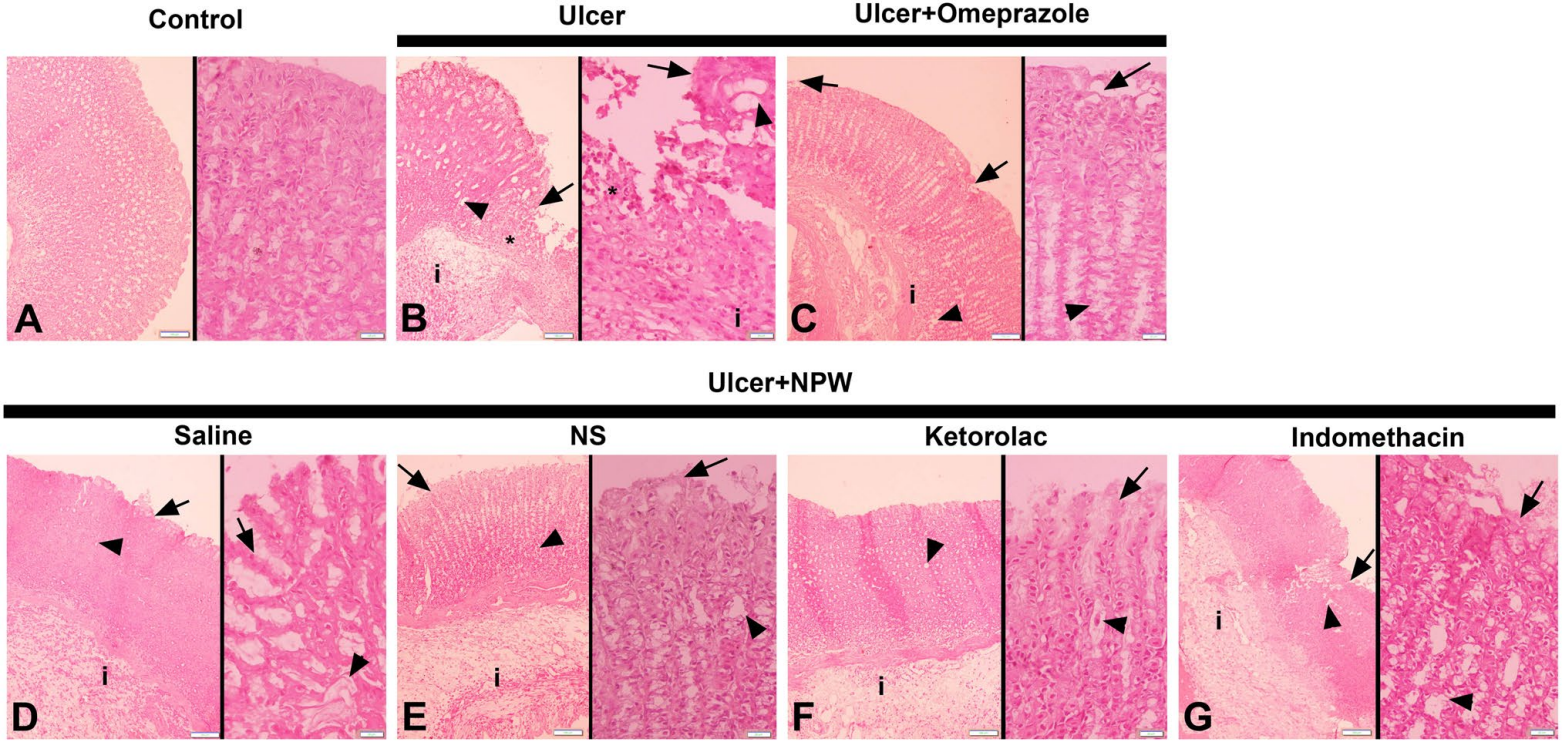
Representative hematoxylin and eosin-stained light micrographs of experimental groups. Acetic acid-induced ulcer groups were treated with either saline (**B**), omeprazole (**C**), Neuropeptide W (NPW) + saline (**D**), NPW + cyclooxygenase (COX)-2 inhibitor NS-398 (**E**), NPW + COX-1 inhibitor ketolorac (**F**) and NPW + non-specific COX-inhibitor indomethacin (G) for 3 days starting with the induction ulcer and were compared with the gastric tissues of control rats (**A**). Micrographs demonstrate severe degeneration of surface (arrow) and glandular epithelium (arrowhead), bleeding in mucosa (*) and inflammatory cell infiltration (**i**). Hematoxylin and Eosin staining. Original magnification and scale bar: Left side: 100 × and 100 µm; right side: 400 × and 20 µm

Histopathological findings revealed that regular mucosa and submucosa observed in the control group were replaced with severe damage in surface and glandular epithelium, severe mucosal hemorrhage, intense inflammatory cell infiltration, and submucosal edema in the saline-treated ulcer group (Fig. 3). In parallel with the biochemical data, high microscopic scores in the saline-treated ulcer group (p < 0.001) were reduced significantly by both omeprazole and NPW treatments (*p* < 0.05; Fig. 2). Mild degeneration in mucous cells and glandular cells, mild to moderate inflammatory cell infiltration and submucosal edema were determined in omeprazole- or NPW-treated ulcer groups (Fig. 3). When either of the selective COX-inhibitors were applied before NPW treatment, a moderate degeneration in mucous cells and moderate degeneration in glandular cells, inflammatory cell infiltration and submucosal edema were identified. In the indomethacin plus NPW-treated ulcer group, a moderate degeneration in mucous and glandular cells with moderate inflammatory cell infiltration and submucosal edema were observed.

**Fig. 4:**
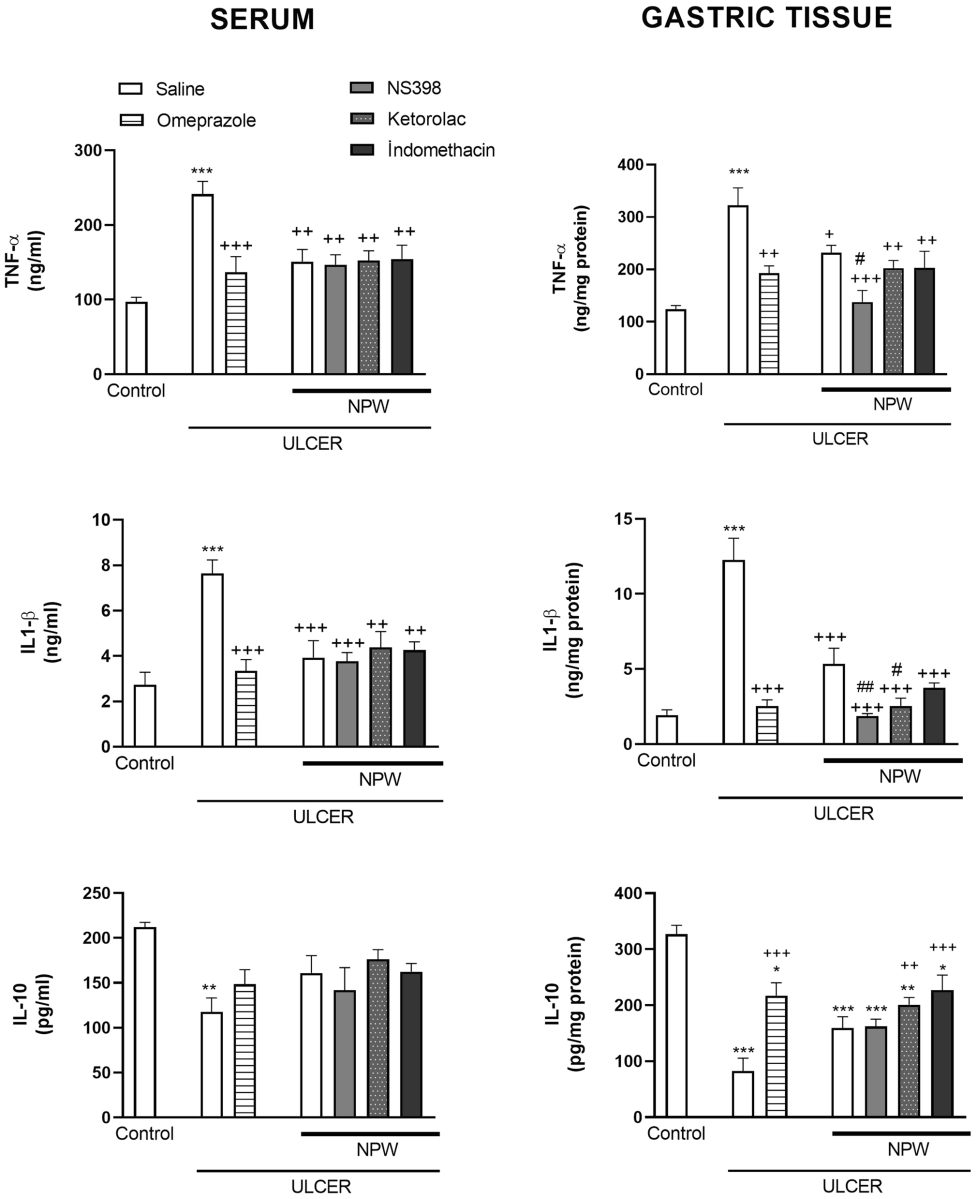
The levels of tumor necrosis factor- alpha (TNF-α), interleukin-1 beta (IL-1β) and IL-10 in the serum and gastric tissues of experimental groups. Acetic acid-induced ulcer groups were treated with either saline, omeprazole, neuropeptide W (NPW) + saline, NPW + cyclooxygenase (COX)-2 inhibitor NS-398, NPW + COX-1 inhibitor ketolorac and NPW + non-specific COX-inhibitor indomethacin for 3 days starting with the induction of acetic acid ulcer and were compared with the gastric tissues of control rats. Each group consisted of 8 rats. ***p < 0.001, compared to control group; ^+^*p* < 0.05, ^++^*p* < 0.01, ^+++^*p* < 0.001 compared to saline-treated ulcer group; ^#^*p* < 0.05, ^##^*p* < 0.01, compared to NPW-treated ulcer group

In the gastric tissue and serum samples of saline-treated ulcer group, levels of the pro-inflammatory cytokines TNF-α and IL-1β were both elevated as compared to control group (*p* < 0.001), while the anti-inflammatory IL-10 level was depressed (*p* < 0.001–0.001; Fig. 4). Omeprazole or NPW treatment significantly reduced the gastric and serum TNF-α and IL-1β levels (*p* < 0.05–0.001). Reductions in serum and gastric IL-10 levels due to ulcer inductions were abolished by both NPW and omeprazole, but elevation in IL-10 was statistically significant only in the gastric tissue of omeprazole-treated ulcer group (*p* < 0.001). None of the COX-inhibitors altered NPW-induced decrease in the serum pro-inflammatory cytokine levels. Gastric TNF-α level was further reduced by NS-398 as compared to NPW-treated ulcer group (*p* < 0.05), while gastric IL-1β levels were depressed more with both of the specific COX-inhibitors (*p* < 0.05–0.01).

**Fig. 5:**
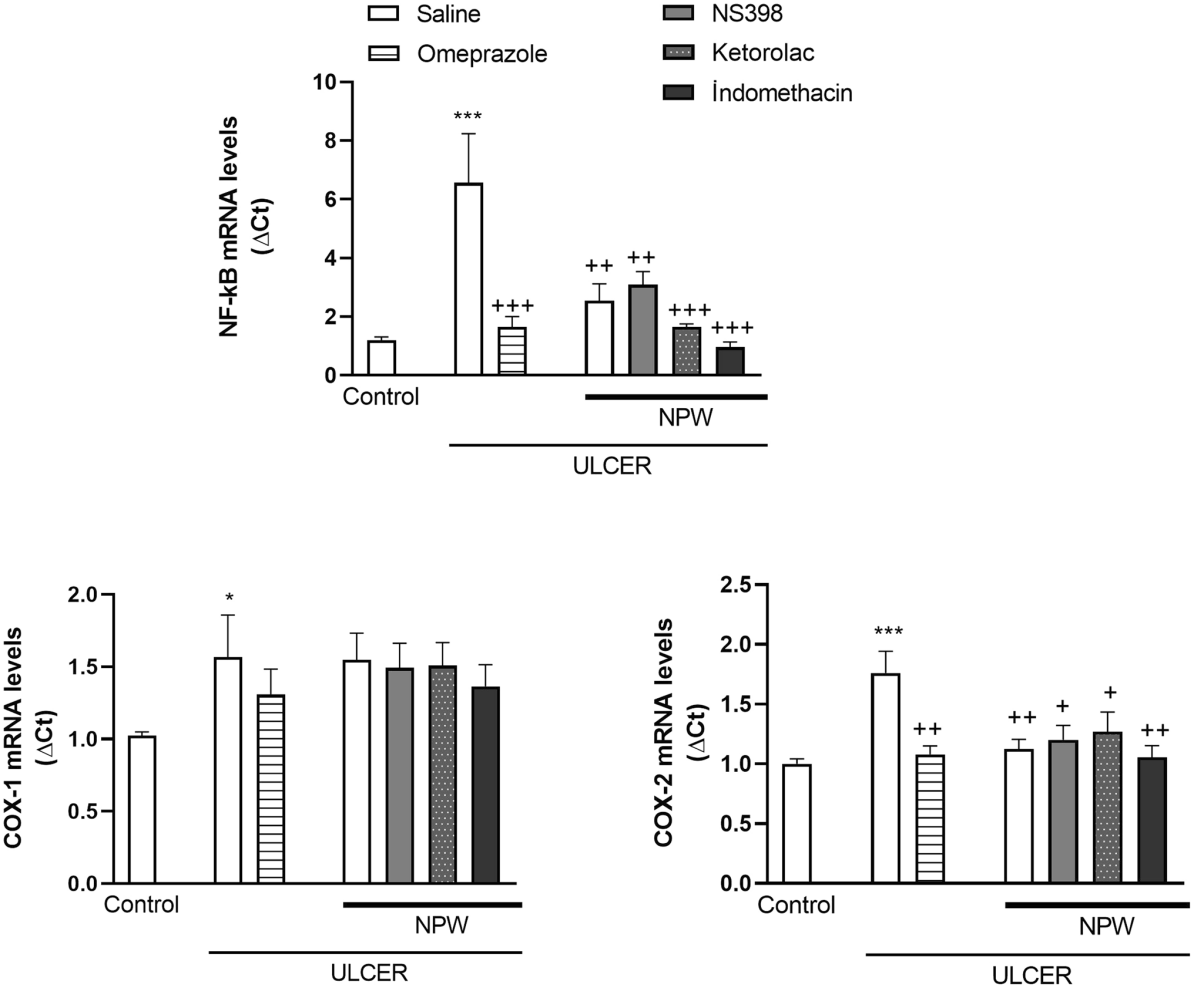
Nuclear factor kappa B (NF-κB), cyclooxygenase (COX)-1 and COX-2 mRNA expression levels of gastric tissues in the experimental groups. Acetic acid-induced ulcer groups were treated with either saline, omeprazole, Neuropeptide W (NPW) + saline, NPW + COX-2 inhibitor NS-398, NPW + COX-1 inhibitor ketolorac and NPW + non-specific COX-inhibitor indomethacin for 3 days starting with the induction of acetic acid ulcer and were compared with the gastric tissues of control rats. Each group consisted of 8 rats. *p < 0.05, ***p < 0.001, compared to control group; ^+^*p* < 0.05, ^++^*p* < 0.01, ^+ + + ^*p* < 0.001 compared to saline-treated ulcer group

In parallel with the changes in cytokine levels, gastric NF-κB expression, which is the mediator of pro-inflammatory gene induction, was also elevated in the saline-treated ulcer group (*p* < 0.001; Fig. 5), and this upregulation of NF-κB expression was depressed by omeprazole or NPW alone or NPW along with any of the COX-inhibitors (*p* < 0.01–0.001). When compared with the control, gastric COX-1 (*p* < 0.05) and COX-2 (*p* < 0.001) protein expressions were significantly upregulated in the saline-treated ulcer group. While omeprazole or NPW treatment significantly suppressed the gastric COX-2 expression (*p* < 0.01; Fig. 5), none of the three COX-inhibitors altered the effect of NPW on COX-2 expression. On the other hand, ulcer-induced elevation in COX-1 protein expression was also continued in the groups treated with either NPW or omeprazole, but the levels of COX-1 protein were not different than that of the control group.

**Fig. 6:**
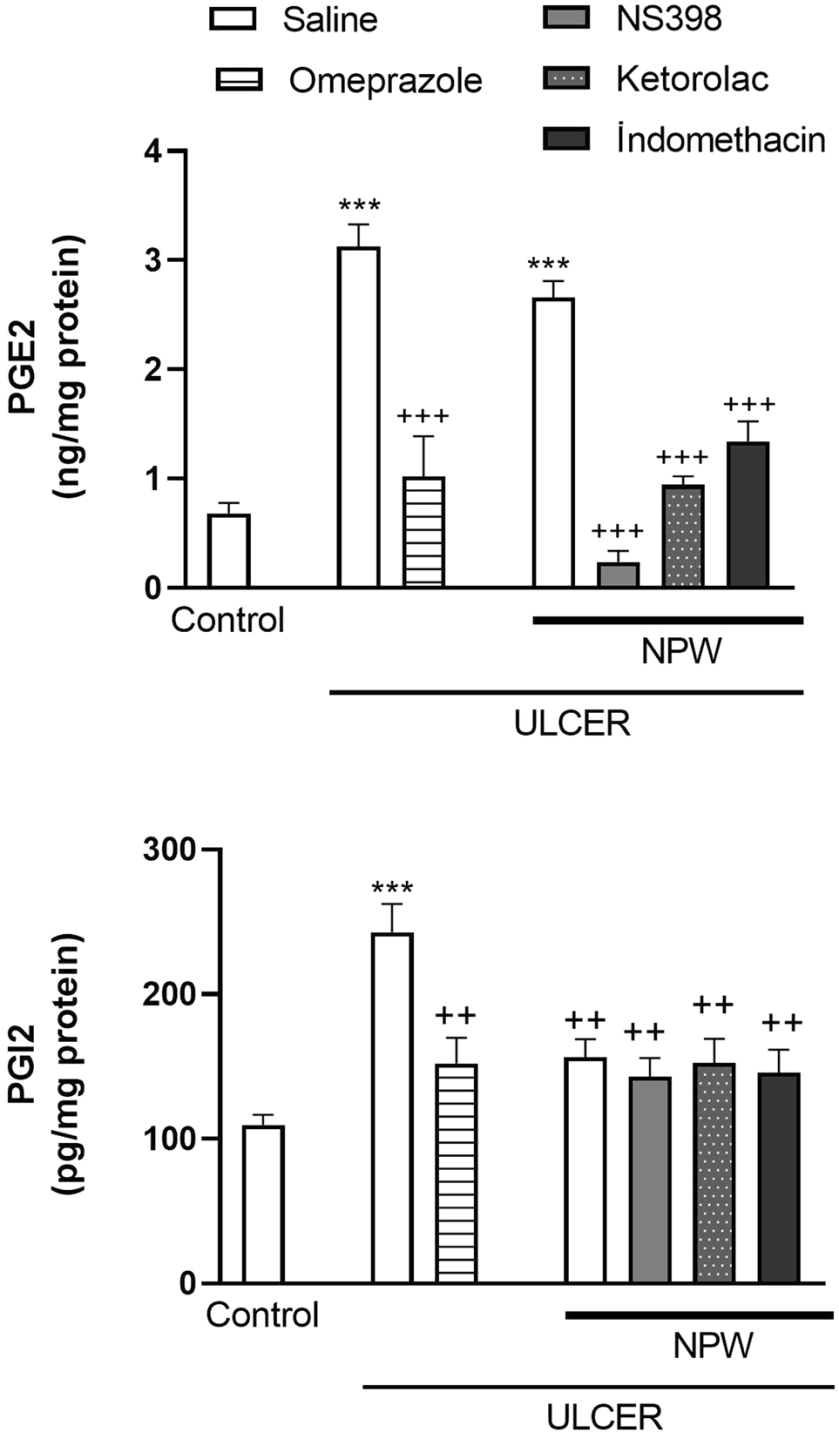
Prostaglandin E2 (PGE2) and prostaglandin I2 (PGI2) levels of gastric tissues in the experimental groups. Acetic acid-induced ulcer groups were treated with either saline, omeprazole Neuropeptide W (NPW) + saline, NPW + cyclooxygenase (COX)-2 inhibitor NS-398, NPW + COX-1 inhibitor ketolorac and NPW + non-specific COX-inhibitor indomethacin for 3 days starting with the induction of acetic acid ulcer and were compared with the gastric tissues of control rats. Each group consisted of 8 rats. ***p < 0.001, compared to control group;^ ++^*p* < 0.01, ^+++ ^*p* < 0.001 compared to saline-treated ulcer group

In the saline-treated ulcer group, gastric levels of PGE2 and PGI2 were significantly increased as compared to non-ulcer control group (*p* < 0.001; Fig. 6). In the omeprazole-treated ulcer group, the elevations in PGE2 and PGI2 were abolished (*p* < 0.01 and *p* < 0.001). Similar to omeprazole, NPW treatment depressed the gastric PGI2 level (*p* < 0.01), while none of the COX-inhibitors altered the NPW-induced decrease in the gastric tissue. Despite that ulcer-induced elevation in PGE2 level was not changed by NPW, all three COX-inhibitors, when given with NPW, significantly reduced gastric PGE2 (*p* < 0.001).

## Discussion

The results of the present study showed that serosal application of acetic acid to induce gastric ulcer has resulted in gastric damage with elevated levels of pro-inflammatory cytokines along with increased neutrophil infiltration, lipid peroxidation, cyclooxygenases and prostaglandins, in conjunction with depleted antioxidant GSH in the gastric tissue. On the other hand, NPW treatment, similar to which occurred with omeprazole treatment, facilitated the healing of gastric injury by suppressing oxidative damage and inflammation along with the replenishment of antioxidant capacity. Moreover, upregulation of gastric COX-1 protein and NF-κB gene due to ulcer induction were abolished by both omeprazole and NPW treatments. On the other hand, COX-1 selective inhibitor, ketorolac, and the non-selective COX-inhibitor, indomethacin -but not the COX-2 inhibitor- partially reversed the NPW-induced alleviation in gastric oxidative injury, indicating that the antioxidant properties of NPW involve a COX-1-dependent mechanism.

Gastric ulcer is a chronic disease characterized by the episodes of relapses that may exist in the same area or recur at another location throughout the patient's life (Kangwan et al. 2014). Similar to human gastric ulcers that become chronic, ulcers induced by acetic acid (Okabe and Amagase 2005; Takagi et al. 1969) occur by changes in multiple factors including prostaglandins, growth factors, nitric oxide, and cytokines, as well as of mucus adhesion pattern and microcirculation (Kobayashi et al. 2001). Ulcer healing consists of various physiological and structural mechanisms, such as angiogenesis and re-epithelialization, as well as oxidative stress and inflammation that include the upregulated activities of cyclooxygenase enzymes and prostaglandins (Kangwan et al. 2014; Yamane et al. 2022). Acetic acid-induced gastric injury is characterized by elevated pro-inflammatory cytokines like TNF-α, IL-1β, and IL-6 in the gastric tissue accompanied by an increase of lipid peroxides and a decrease of antioxidant capacity (Ajeigbe et al. 2022; Eamlamnam et al. 2006; Xue et al. 2019), making suppression of oxidative stress and inflammatory process a main target for the healing process of gastric ulcer. In accordance with the literature, the current results also demonstrated that neutrophil infiltration, lipid peroxidation, pro-inflammatory cytokine release, and upregulation of COX enzymes and prostaglandins are evident in the acetic acid-induced gastric ulcer. Our results also showed that NPW treatment inhibited lipid peroxidation and MPO activity, reduced release of pro-inflammatory cytokines and replenished the antioxidant GSH content, showing that the anti-ulcer effect of NPW occurs by inhibiting neutrophil recruitment and upregulating the antioxidant capacity during the chronic oxidative progression of gastric ulcer. Stimulation of NF-κB, which plays an important role in the development and progression of gastric ulcer, controls the magnitude of the inflammatory response in the gastric mucosa by modulating the production of a variety of cytokines and chemokines (Marta et al. 2020; Yeo et al. 2018). Accordingly, the agents that exert anti-inflammatory effects in acetic acid-induced gastric ulcer act by the inhibition of NF-κB (Konturek et al. 2008). We have previously reported that NPW improved sepsis-induced multiple organ injury and stress-induced gastric injury by suppressing oxidative stress via the inhibition of NF-κB signaling (Atici et al. 2022; Tamer et al. 2022). In the present study, the serum or gastric pro-inflammatory cytokines TNF-α and IL-1β as well as the mRNA expression of NF-κB were reduced by NPW treatment in the rat gastric tissue, indicating the anti-inflammatory action of NPW against acetic acid-induced gastric injury also occurs through the suppression of NF-κB pathway.

It is well known that gastric and intestinal lesions develop by the inhibition of either COX-1 and COX-2 activity by COX-inhibitors (Halter et al. 2001). Although COX-2 appears as the key mediator in gastric wound healing, COX-1 becomes important when COX-2 is impaired (Schmassmann et al. 2006). Our current findings revealed that blockade of COX-1 enzyme as well as treatment with the non-specific COX-inhibitor indomethacin reversed the effects of NPW on neutrophil recruitment and antioxidant capacity, suggesting that COX-1 enzyme activity mediates the therapeutic effect of NPW on gastric oxidative damage. The primary functions of COX-1 are to maintain the integrity of the gastric mucosa and to control gastric blood flow and acid secretion, whereas COX-2 enzyme activated during an inflammatory response upregulates prostaglandin synthesis at the location of ulceration (Mizuno et al. 1997; Ricciotti and FitzGerald 2011). During both the acute inflammation phase and ulcer healing phase, COX-1 and COX-2 expressions are upregulated at the area of the gastric ulcer, particularly in areas of extensive tissue repair (Rodríguez et al. 2003). Previous studies showed that COX-1 and COX-2 was upregulated in *H. pylori* gastritis, especially the mid glandular zone and lamina propria inflammatory cells in the human gastric mucosa (Jackson et al. 2000), and also acetic acid-induced gastric ulcer model (Fagundes et al. 2020; Kolgazi et al. 2017). In agreement with these studies, our results confirmed that gastric COX-1 and COX-2 mRNA levels are elevated as a compensatory mechanism in response to a 3-day chronic gastric ulcer. On the other hand, NPW treatment depressed the degree of lipid peroxidation, neutrophil infiltration and pro-inflammatory cytokine levels, and elevated antioxidant capacity in conjunction with the downregulation of gastric COX-1 and COX-2 mRNA levels, suggesting that improved gastric damage due to NPW could be eliminating the need for compensatory upregulation of COX enzymes. Moreover, the suppressive effect of NPW on oxidative injury was reversed by selective blockade of COX-1, showing the specific role of COX-1 enzyme activity in the protective effect of NPW, which was comparable to that of omeprazole treatment. Although we have previously reported that the protective effect of NPW against stress-induced gastric ulcer and acetic acid-induced colonic injury involves its modulatory effect on the COX enzyme system and cytokine production (Arabacı Tamer et al. 2023; Tamer et al. 2022), the involvement of COX enzymes in the healing-promoting effects of NPW in gastric ulcer was not identified before. Thus, the current study emphasizes that the protective effect of NPW against acute oxidative gastric injury as well as its facilitatory action in chronic healing of gastric ulcer involves its modulatory role in the activity of COX enzyme system.

Prostaglandins generated by particularly the COX-2 enzyme, have a key role in ulcer healing process by increasing blood flow, stimulating mucosal bicarbonate secretion, preventing the disruption of mucosal barrier, accelerating cell proliferation and enhancing angiogenesis (Poonam et al. 2005; Takeuchi and Amagase 2018). Kolgazi et al. (2017) have demonstrated that blockade of selective COX-1 or COX-2 enzymes or non-selective COX-inhibitor indomethacin, reversed most of the therapeutic effects of nesfatin-1 peptide on acetic acid-induced gastric ulcer, while COX-2-blockade was consistently more effective. It has been reported that NSAIDs reduce PGE2 content in the gastric tissue and lead to significantly delayed chronic gastric ulcer healing in rats (Berenguer et al. 2002). Additionally, gastric PGE2 level was found to be elevated in parallel with the occurrence of oxidative stress and inflammation in acetic acid-induced gastric ulcer model (Ercan et al. 2020; Mohammadifard et al. 2021). Thus, PG levels, which are known to protect the gastric mucosa by increasing blood flow, were not altered by NPW treatment, while blockade of COX enzymes reduced the PG levels. Our previous results demonstrated that NPW treatment prevented reduction in blood flow during the chronic colitis and gastric ulcer by modulating COX enzyme system (Arabacı Tamer et al. 2023; Tamer et al. 2022). Since NPW receptors are widely expressed in the peripheral tissues that include blood vessels (Chottova Dvorakova 2018), NPW may have a regulatory function in the maintenance of gut microcirculation without affecting prostaglandins in the acetic acid-induced gastric ulcer, which may contribute to the ameliorative effect of NPW treatment in chronic gastric ulcer.

In conclusion, the present data revealed that the intraperitoneal administration of NPW improves gastric injury in rats via the inhibition of the pro-inflammatory cytokine production, oxidative stress and inflammation as well as the downregulation of COX-2 protein and NF-κB gene expressions. Our data indicates that additional experimental and clinical studies are necessary to explore the potential application of NPW for the enhancement of chronic gastric ulcer healing. It would be possible to learn more about the endogenous function of NPW and its interaction with the COX enzymes during the healing of gastric ulcers if specific NPW antagonists were to be developed.

## Data Availability

Data will be made available on request.

## References

[CR1] Ajeigbe K, Aibangbee K, Saeed S, Ajeigbe OOnifade A, (2022). Folic acid protects and heals gastric mucosa: role of acid output, inflammatory cytokines, angiogenic and growth factors. J Basic Appl Zool.

[CR2] Arabacı Tamer S, Akbulut S, Erdoğan Ö, Çevik Ö, Ercan FYeğen BÇ (2023) Neuropeptide W Exhibits Preventive and Therapeutic Effects on Acetic Acid-Induced Colitis via Modulation of the Cyclooxygenase Enzyme System. Digestive Diseases and Sciences, 1–13 10.1007/s10620-022-07811-2 36631709

[CR3] Arabacı Tamer S, Üçem S, Büke B, Güner M, Karaküçük AG, Yiğit N (2020). Regular moderate exercise alleviates gastric oxidative damage in rats via the contribution of oxytocin receptors. J Physiol.

[CR4] Arabaci Tamer S, YILDIRIM A, ÇEVİK Ö, AKSU M, YÜKSEL M, Dertsiz E, (2021). The ameliorative effects of melatonin on acetic acid-induced gastric ulcer in rats via its modulatory effects on gut microbiota. Cukurova Med J.

[CR5] Atici AE, Arabacı Tamer S, Levent HN, Peker Eyüboğlu İ, Ercan F, Akkiprik M (2022). Neuropeptide W attenuates oxidative multi-organ injury in rats induced with intra-abdominal sepsis. Inflammation.

[CR6] Bereda G (2022). Peptic Ulcer disease: definition, pathophysiology, and treatment. J Biomed Biol Sci.

[CR7] Berenguer B, de la Lastra CA, Moreno FJMartín MJ, (2002). Chronic gastric ulcer healing in rats subjected to selective and non-selective cyclooxygenase-2 inhibitors. Eur J Pharmacol.

[CR8] Chottova Dvorakova M (2018). Distribution and function of neuropeptides W/B signaling system. Front Physiol.

[CR9] Eamlamnam K, Patumraj S, Visedopas NThong-Ngam D, (2006). Effects of Aloe vera and sucralfate on gastric microcirculatory changes, cytokine levels and gastric ulcer healing in rats. World J Gastroenterol: WJG.

[CR10] Ercan G, Tartar RI, Solmaz A, Gulcicek OB, Karagulle OO, Meric S (2020). Potent therapeutic effects of ruscogenin on gastric ulcer established by acetic acid. Asian J Surg.

[CR11] Fagundes FL, Piffer GdM, Périco LL, Rodrigues VP, Hiruma-Lima CADos Santos RdC, (2020). Chrysin modulates genes related to inflammation, tissue remodeling, and cell proliferation in the gastric ulcer healing. Int J Mol Sci.

[CR12] Fujii R, Yoshida H, Fukusumi S, Habata Y, Hosoya M, Kawamata Y (2002). Identification of a neuropeptide modified with bromine as an endogenous ligand for GPR7. J Biol Chem.

[CR13] Halter F, Tarnawski A, Schmassmann APeskar B, (2001). Cyclooxygenase 2—implications on maintenance of gastric mucosal integrity and ulcer healing: controversial issues and perspectives. Gut.

[CR14] Jackson L, Wu K, Mahida Y, Jenkins DHawkey C, (2000). Cyclooxygenase (COX) 1 and 2 in normal, inflamed, and ulcerated human gastric mucosa. Gut.

[CR15] Ji L, Zhu H, Chen H, Fan W, Chen J, Chen J (2015). Modulation of CaV1. 2 calcium channel by neuropeptide W regulates vascular myogenic tone via G protein-coupled receptor 7. J Hypertens.

[CR16] Kangwan N, Park J-M, Kim E-HHahm KB, (2014). Quality of healing of gastric ulcers: natural products beyond acid suppression. World J Gastrointest Pathophysiol.

[CR17] Kobayashi T, Ohta Y, Yoshino J, Nakazawa S (2001). Teprenone promotes the healing of acetic acid-induced chronic gastric ulcers in rats by inhibiting neutrophil infiltration and lipid peroxidation in ulcerated gastric tissues. Pharmacol Res.

[CR18] Kolgazi M, Ozdemir-Kumral Z, Cantali-Ozturk C, Demirci E, Yuksel M, Sirvanci S (2017). Anti-inflammatory effects of nesfatin-1 on acetic acid-induced gastric ulcer in rats: involvement of cyclo-oxygenase pathway. J Physiol Pharmacol.

[CR19] Konturek P, Burnat G, Brzozowski T, Zopf YKonturek S, (2008). Tryptophan free diet delays healing of chronic gastric ulcers in rat. J Physiol Pharmacol.

[CR20] Laine L, Takeuchi K, Tarnawski A (2008). Gastric mucosal defense and cytoprotection: bench to bedside. Gastroenterology.

[CR21] Marta Ż-N, Agnieszka W, Jeleń A, Adrian K, Dagmara S-K, Sałagacka-Kubiak A (2020). NFKB2 gene expression in patients with peptic ulcer diseases and gastric cancer. Mol Biol Rep.

[CR22] Mizuno H, Sakamoto C, Matsuda K, Wada K, Uchida T, Noguchi H (1997). Induction of cyclooxygenase 2 in gastric mucosal lesions and its inhibition by the specific antagonist delays healing in mice. Gastroenterology.

[CR23] Mohammadifard M, Javdani H, Khalili-Tanha G, Farahi A, Foadoddini MHosseini M (2021) Evaluation of Therapeutic Efficacy of Stigma and Petal Extracts of *Crocus sativus* L. on Acetic Acid-Induced Gastric Ulcer in Rats. Traditional and Integrative Medicine.

[CR24] Mondal MS, Yamaguchi H, Date Y, Shimbara T, Toshinai K, Shimomura Y (2003). A role for neuropeptide W in the regulation of feeding behavior. Endocrinology.

[CR25] Morita I (2002). Distinct functions of COX-1 and COX-2. Prostaglandins Other Lipid Mediat.

[CR26] Narayanan M, Reddy KM, Marsicano E (2018). Peptic ulcer disease and Helicobacter pylori infection. Missouri Med.

[CR27] Niimi M, Murao K (2005). Neuropeptide W as a stress mediator in the hypothalamus. Endocrine.

[CR28] Okabe S, Amagase K (2005). An overview of acetic acid ulcer models—the history and state of the art of peptic ulcer research. Biol Pharmac Bull.

[CR29] Okabe S, Roth JL, Pfeiffer CJ (1971). A method for experimental, penetrating gastric and duodenal ulcers in rats. Am J Digest Dis.

[CR30] Oyetayo NS, Kodie DO, Nwakasi MI, Afolabi OO, Jarikre TA, Eyarefe OD (2022). Gastroprotective and ulcer healing potentials of Nigerian Bee Propolis flavonoid extract on acetic acid-induced gastric ulcers in albino rats (Wistar Strains). Adv Trad Med.

[CR31] Pate AT, Yosten GL, Samson WK (2013). Neuropeptide W increases mean arterial pressure as a result of behavioral arousal. Am J Physiol Regul Integr Comp Physiol.

[CR32] Poonam D, Vinay CS, Gautam P (2005). Cyclo-oxygenase-2 expression and prostaglandin E2 production in experimental chronic gastric ulcer healing. Eur J Pharmacol.

[CR33] Ricciotti E, FitzGerald GA (2011). Prostaglandins and inflammation. Arterioscl Thromb Vasc Biol.

[CR34] Rodríguez JA, Astudillo L, Schmeda-Hirschmann G (2003). Oleanolic acid promotes healing of acetic acid-induced chronic gastric lesions in rats. Pharmacol Res.

[CR35] Salari N, Darvishi N, Shohaimi S, Bartina Y, Ahmadipanah M, Salari HR (2022). The global prevalence of peptic ulcer in the world: a systematic review and meta-analysis. Indian J Surg.

[CR36] Schmassmann A, Zoidl G, Peskar BM, Waser B, Schmassmann-Suhijar D, Gebbers J-O (2006). Role of the different isoforms of cyclooxygenase and nitric oxide synthase during gastric ulcer healing in cyclooxygenase-1 and-2 knockout mice. Am J Physiol.

[CR37] Shimomura Y, Harada M, Goto M, Sugo T, Matsumoto Y, Abe M (2002). Identification of neuropeptide W as the endogenous ligand for orphan G-protein-coupled receptors GPR7 and GPR8. J Biol Chem.

[CR38] Sverdén E, Agréus L, Dunn JM, Lagergren J (2019). Peptic ulcer disease. BMJ.

[CR39] Tache Y, Perdue M (2004). Role of peripheral CRF signalling pathways in stress-related alterations of gut motility and mucosal function. Neurogastroenterol Motility.

[CR40] Takagi K, Okabe-Ssaziki R (1969). A new method for the production of chronic gastric ulcer in rats and the effect of several drugs on its healing. Jap J Pharmacol.

[CR41] Takenoya F, Kageyama H, Hirako S, Ota E, Ogawa T, Wada N (2012). Neuropeptide w. Front Endocrinol.

[CR42] Takeuchi K, Amagase K (2018). Roles of cyclooxygenase, prostaglandin E2 and EP receptors in mucosal protection and ulcer healing in the gastrointestinal tract. Curr Pharm Des.

[CR43] Tamer SA, Akbulut S, Eyüboğlu İP, Erdoğan Ö, Çevik Ö, Akkiprik M (2022). Peripheral administration of Neuropeptide-W protects against stress-induced gastric injury in rats. Life Sci.

[CR44] Tuğtepe H, Şener G, Bıyıklı NK, Yüksel M, Çetinel Ş, Gedik N (2007). The protective effect of oxytocin on renal ischemia/reperfusion injury in rats. Regul Pept.

[CR45] Wang X-Y, Wang M, Yin J-Y, Song Y-H, Wang Y-X, Nie S-P (2022). Gastroprotective activity of polysaccharide from the fruiting body of Hericium erinaceus against acetic acid-induced gastric ulcer in rats and structure of one bioactive fraction. Int J Biol Macromol.

[CR46] Xue Z, Shi G, Fang Y, Liu X, Zhou X, Feng S (2019). Protective effect of polysaccharides from Radix Hedysari on gastric ulcers induced by acetic acid in rats. Food Funct.

[CR47] Yamane S, Amano H, Ito Y, Betto T, Matsui Y, Koizumi W (2022). The role of thromboxane prostanoid receptor signaling in gastric ulcer healing. Int J Exp Pathol.

[CR48] Yandrapu H, Sarosiek J (2015). Protective factors of the gastric and duodenal mucosa: an overview. Curr Gastroenterol Rep.

[CR49] Yeo D, Hwang SJ, Kim WJ, Youn H-JLee H-J, (2018). The aqueous extract from Artemisia capillaris inhibits acute gastric mucosal injury by inhibition of ROS and NF-kB. Biomed Pharmacother.

[CR50] Yu N, Chu C, Kunitake T, Kato K, Nakazato MKannan H, (2007). Cardiovascular actions of central neuropeptide W in conscious rats. Regul Pept.

[CR51] Zatorski H (2017). Pathophysiology and risk factors in peptic ulcer disease. Intr Gastroint Dis.

[CR52] Zewdu WS, Aragaw TJ (2020). Evaluation of the anti-ulcer activity of hydromethanolic crude extract and solvent fractions of the root of Rumex nepalensis in Rats. J Exp Pharmacol.

